# Cationic Complexes of Hydrogen with Helium

**DOI:** 10.1002/cphc.201200664

**Published:** 2012-10-22

**Authors:** Peter Bartl, Christian Leidlmair, Stephan Denifl, Paul Scheier, Olof Echt

**Affiliations:** [a] Institut für Ionenphysik und Angewandte Physik, Universität InnsbruckTechnikerstraße 25, A-6020 Innsbruck (Austria); [b] Department of Physics, University of New HampshireDurham, NH 03824 (USA)

**Keywords:** clusters, helium, hydrides, ion–molecule reactions, mass spectrometry

## Abstract

High-resolution mass spectra of helium nanodroplets doped with hydrogen or deuterium reveal that copious amounts of helium can be bound to H^+^, H_2_^+^, H_3_^+^, and larger hydrogen-cluster ions. All conceivable He_*n*_H_*x*_^+^ stoichiometries are identified if their mass is below the limit of ≍120 u set by the resolution of the spectrometer. Anomalies in the ion yields of He_*n*_H_*x*_^+^ for *x*=1, 2, or 3, and *n*≤30 reveal particularly stable cluster ions. Our results for He_*n*_H_1_^+^ are consistent with conclusions drawn from previous experimental and theoretical studies which were limited to smaller cluster ions. The He_*n*_H_3_^+^ series exhibits a pronounced anomaly at *n*=12 which was outside the reliable range of earlier experiments. Contrary to findings reported for other diatomic dopant molecules, the monomer ion (i.e. H_2_^+^) retains helium with much greater efficiency than hydrogen-cluster ions.

## 1. Introduction

Recent attempts to synthesize compounds that contain noble gases have been remarkably successful.^1^–^3^ However, so far no stable compound containing helium has been synthesized, except for structures where helium atoms are physically trapped inside fullerene cages.^4^ One promising type of novel compounds are noble-gas hydrides of the form HNgY where Ng is a noble-gas atom and Y an electronegative fragment.^5^, ^6^ A recent review^2^ lists 23 of these hydrides, but none including helium. HHeF was initially predicted to be merely metastable.^7^ It might in fact be stabilized by the environment but has not yet been detected.^6^, ^8^

On the other hand, many atomic and molecular ions bind to helium in the gas phase (see the reviews by Grandinetti^3^, ^9^). The simplest of these complexes, HeH^+^, was first observed in a mass spectrum by Hogness and Lunn.^10^ The ion has a calculated bond length of 0.77 Å and a dissociation energy of 1.844 eV.^11^ The endothermic proton transfer reaction [Eq. (1)]:

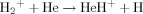
1

is a prototype ion–molecule reaction that has been studied in great detail by experimentalists and theorists alike (see ref. 12 and references therein).

Protonated helium clusters He_*n*_H^+^ have been the subject of numerous theoretical studies.^11^, ^13^–^17^ He_2_H^+^ has been observed to form in collisions between He_2_^+^ and H_2_ in a flowing afterglow.^18^ Its calculated dissociation energy into HeH^+^+He is about 0.56 eV (see ref. 9 and references therein). The linear, centrosymmetric (point group *D*_∞h_) He_2_H^+^ ion forms the chromophore in larger He_*n*_H^+^ complexes.^15^–^17^

As hydrogen and helium are the most abundant elements in the universe it is conceivable that condensation of helium at molecular hydrogen ions plays a role in the chemistry of astronomical objects as well. Hogness and Lunn^10^ already observed a weak signal at 6 u that they assigned to HeH_2_^+^. The calculated dissociation energy of this ion is about 0.21 eV.^9^ The existence of a long-lived, electronically excited He_2_H_2_^+^ ion with centrosymmetric structure, [He-H-H-He]^+^, was proposed by Uggerud and co-workers^19^ and experimentally identified based on its unusual metastable dissociation into HeH^+^+neutral fragments.^20^ To the best of our knowledge, no calculations exist for complexes of H_2_^+^ with more than two helium atoms.

H_3_^+^, the most abundantly produced interstellar molecular ion,^21^ may be a trap for helium and other noble gases in the universe.^22^, ^23^ Chakraborty et al. have reported a density functional theory study of He_*n*_H_3_^+^ (*n*≤3),^24^ but larger complexes have not yet been considered.

This brief review of the literature documents considerable interest in the properties of cationic complexes of hydrogen with helium. However, we are aware of only two experimental reports that involve more than two helium atoms. Kojima et al.^25^ observed He_*n*_H^+^ (*n*≤14) by injecting H_2_^+^ into a drift tube filled with helium at 4.4 K. Injection of H_3_^+^ gave rise to He_*n*_H^+^ (*n*≤14) and He_*n*_H_3_^+^ (*n*≤13). Only a very weak signal of He_*n*_H_2_^+^ (3≤*n*≤10) could be observed. They measured the drift-field dependence of the ion yield and concluded that He_6_H^+^, He_13_H^+^, He_9_H_3_^+^, and He_10_H_3_^+^ are particularly stable.

The second experimental study, by Toennies and co-workers,^26^ utilized the technique to grow atomic or molecular clusters in ultracold superfluid helium nanodroplets.^27^, ^28^ Excess energy released during cluster formation and subsequent ionization of the dopant was quickly removed from the nascent ion by the evaporation of helium. Mass spectra demonstrated the solvation of Ne^+^, Ar^+^, Kr^+^ and Xe^+^,^29^, ^30^ and many other atomic, molecular, and cluster ions^31^–^33^ in helium. In the study by Toennies and co-workers,^26^ the nanodroplets were doped with deuterium and subsequently ionized by electron impact. Two series of ion peaks were observed, one at mass 4 *n* u (*n*=integer) and the other at 2+4 *n* u. The peaks were assigned to He_*n*_^+^ and He_*n*_D^+^, respectively. Unfortunately, the mass resolution of Δ*m*≍1 u made it impossible to identify contributions from He_*n*_D_2_^+^ or He_*n*_D_3_^+^ which have the same nominal mass as He_*n*+1_^+^ and He_*n*+1_D^+^. Except for a strong peak at 6 u that was assigned to D_3_^+^, the yield of He_*n*_D_2_^+^ or He_*n*_D_3_^+^ was supposed to be negligible.

In a recent study of hydrogen clusters embedded in helium nanodroplets we observed mixed He_*n*_H_*x*_^+^ ions, but the resolution did not suffice to identify mixed ions beyond a mass of ≍23 u.^34^ Herein, we present an investigation of helium–hydrogen and helium–deuterium complexes at improved mass resolution. He_*n*_H_*x*_^+^ and He_*n*_D_*x*_^+^ ions are formed by electron impact and mass-resolved up to about 120 u. For a given nominal mass, all combinations of *n* and *x* that are consistent with that mass are observed. For example, in experiments with H_2_ 15 mass peaks are resolved at a nominal mass of 56 u. They correspond to He_14_^+^, He_13_H_4_^+^, He_12_H_8_^+^,…, H_56_^+^. The ion series He_*n*_H^+^, He_*n*_H_2_^+^, and He_*n*_H_3_^+^ are analyzed in detail. For a given value of *n* their yields are of the same order of magnitude. Anomalies in the distribution of the ion yield versus *n* are attributed to anomalies in ion stability. In agreement with earlier experiments by Kojima et al.^25^ the He_*n*_H^+^ series reveals enhanced stability of He_13_H^+^. However, contrary to Kojima et al. we find evidence for enhanced stability of He_12_H_3_^+^ rather than He_9_H_3_^+^ and He_10_H_3_^+^. The He_*n*_H_2_^+^ series shows a weak anomaly at *n*=19. By and large, experiments with D_2_ corroborate our findings from experiments with H_2_.

## Experimental Section

Neutral helium nanodroplets were produced by expanding helium (purity 99.9999 %) from a stagnation pressure of 2 MPa through a 5 μm nozzle, cooled to 8 to 9 K by a closed-cycle refrigerator (Sumitomo Heavy Industries LTD, model RDK-415D), into vacuum. The estimated average number of helium atoms per droplet formed in the expansion is about 10^6^; the droplets are superfluid with a temperature of ≍0.37 K.^27^ The resulting supersonic beam was skimmed by a 0.8 mm conical skimmer, located 8 mm downstream from the nozzle. The skimmed beam traversed a 20 cm long differentially pumped pickup region into which hydrogen (Messer Austria GmbH, purity 99.999 %) or deuterium (purity 99.7 % by weight) was introduced. The measured partial pressure was about 10^−3^ Pa (uncorrected gauge signal).

After the pickup region the doped helium droplets passed a region in which they were ionized by electron impact at 70 eV. Cations were accelerated to 40 eV into the extraction region of a commercial time-of-flight mass spectrometer equipped with a reflectron (Tofwerk AG, model HTOF); its mass resolution is about Δ*m*/*m*=1/5000. The base pressure in the mass spectrometer is 10^−5^ Pa. The ions were extracted at 90° into the field-free region of the spectrometer by a pulsed extraction voltage. At the end of the field-free region they entered a two-stage reflectron which reflected them towards a microchannel plate detector operated in single-ion counting mode. Additional experimental details have been described elsewhere.^33^, ^35^

## 2. Results

**Figure 1:**
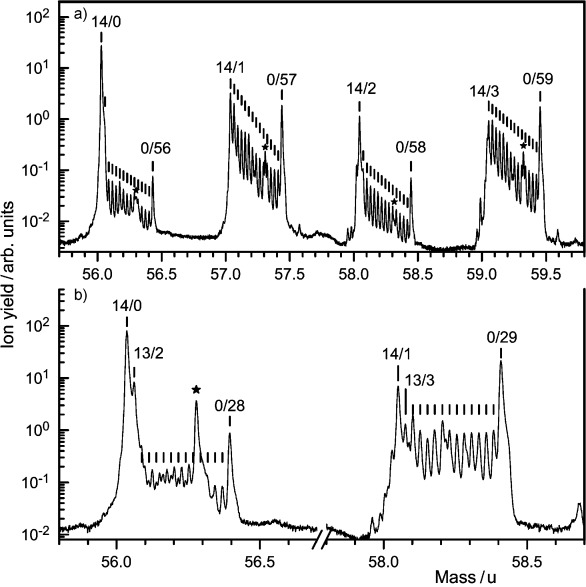
Mass spectra of helium droplets doped with hydrogen (a) or deuterium (b). Vertical lines mark the predicted positions of all He_*n*_H_*x*_^+^ or He_*n*_D_*x*_^+^ ion peaks that could possibly appear. For some peaks the values of *n* and *x* are indicated in the form *n*/*x*. Stars mark cluster ions that contain a H_2_O impurity.

Sections of mass spectra recorded with H_2_-doped and D_2_-doped helium droplets are displayed in Figure 1 a and b. Four groups of mass peaks are seen in Figure 1 a. They correspond to He_14−*m*_H_4 *m*_^+^ at a nominal mass of 56 u, He_14−*m*_H_4 *m*+1_^+^ at 57 u, He_14−*m*_H_4 *m*+2_^+^ at 58 u, and He_14−*m*_H_4 *m*+3_^+^ at 59 u, with 0≤*m*≤14 within each group. The separation between adjacent mass peaks in a group, 0.0287 u, agrees with the mass difference between 4 H and ^4^He.^36^ The resolving power is sufficient to resolve individual mass peaks up to about 120 u. The values of *n* and *x* are indicated as *n*/*x* above some peaks. Vertical lines indicate the positions of all He_*n*_H_*x*_^+^ ions that could possibly appear in the mass range shown. The ions were in fact observed although a few were masked by a water contamination, marked by an asterisk. The mass range below the water contamination in the He_14−*m*_D_2 *m*_^+^ ion series at nominal mass 56 u is dominated by an ion series of unknown origin, probably caused by a contaminant in the D_2_ gas whose purity was only 99.7 % by weight.

In our earlier work involving pure hydrogen-cluster ions^34^ we had already noticed that even-numbered H_*x*_^+^ cluster ions appear with a yield of typically 4 % relative to adjacent odd-numbered cluster ions. This is readily seen in Figure 1 a for H_56_^+^ through H_59_^+^ thanks to the five-fold improvement in mass resolution. More generally, mass peaks in the second and fourth group (containing ions with an odd number of hydrogen atoms) are roughly an order of magnitude more abundant than mass peaks in the first and third group, save for the pure helium peak. In other words, cluster ions He_*n*_H_*x*_^+^ with *x* odd are about an order of magnitude more abundant than even-numbered ones, if one compares ions with approximately equal values of *x*.

A spectrum of He_*n*_D_*x*_^+^ is shown in Figure 1 b. Ions in the first group (nominal mass 56 u) correspond to He_14−*m*_D_2 *m*_^+^ and ions in the second group (58 u) to He_14−*m*_D_2 *m*+1_^+^, with 0≤*m*≤14 within each group. The separation between adjacent mass peaks equals 0.0256 u, the mass difference between 2D and ^4^He.^36^ It is more problematic to extract the yield of ions with a D_2_^+^ or D_3_^+^ core from these spectra because their mass peaks are preceded by prominent peaks due to pure He_*n*_^+^ or ions with a D_1_^+^ core. Nevertheless, the data provides a consistency check for measurements with hydrogen.

**Figure 2:**
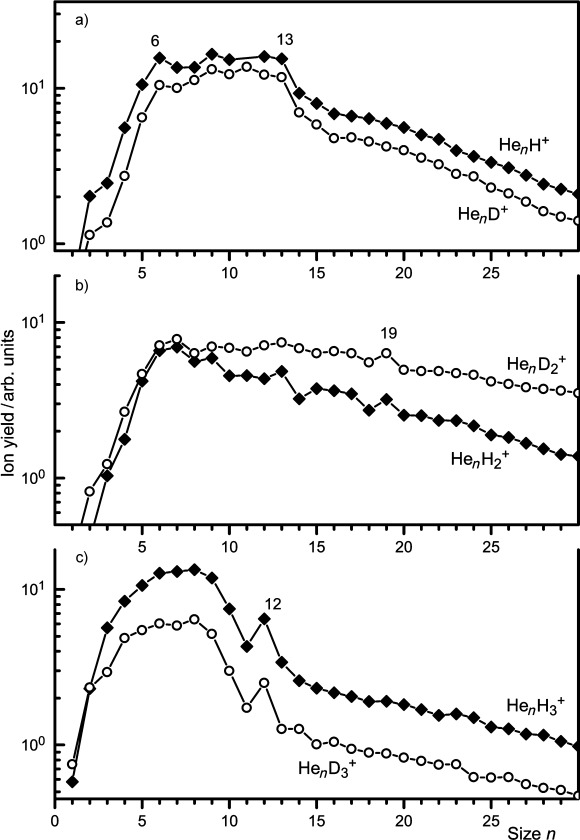
Ion yields of He_*n*_H^+^ (a), He_*n*_H_2_^+^ (b), and He_*n*_H_3_^+^ (c) versus *n*, together with the corresponding yields of He_*n*_D^+^, He_*n*_D_2_^+^, and He_*n*_D_3_^+^. Anomalies in the ion yields that are deemed significant are marked by the value of *n*.

The ion yield of He_*n*_H_*x*_^+^ and He_*n*_D_*x*_^+^ (*x*=1, 2, 3) versus *n* is compiled in Figure 2. A few data points that suffer from contamination (as judged from an analysis of the peak shape and comparison with spectra recorded with undoped helium droplets) have been omitted. Discarding these points we see very close agreement between the data obtained for He_*n*_H_*x*_^+^ and He_*n*_D_*x*_^+^ for *x*=1 and 3. In particular, for *x*=1 the ion yield increases rapidly with *n* until a plateau is reached at *n*=6. An abrupt drop occurs after *n*=13. For *x*=3 the yield increases more quickly to reach a plateau at *n*=4 or 5. A pronounced local maximum appears at *n*=12.

The situation is not as clear for *x*=2. He_*n*_H_2_^+^ features an abrupt drop from *n*=13 to 14 but the He_*n*_D_2_^+^ ion series is essentially flat in this range. In all likelihood one of the two distributions is affected by a contamination but we cannot say for sure which one. However, a weak local maximum occurs for both ion series at *n*=19; it is probably significant.

An analysis of mixed cluster ions containing more than three hydrogen atoms did not reveal any statistically significant abundance anomalies. Increased scatter in the size dependence of the ion abundance and larger discrepancies between He_*n*_H_*x*_^+^ and He_*n*_D_*x*_^+^ indicated contamination by unidentified impurities.

## 3. Discussion

Herein, ions were produced by the interaction of 70 eV electrons with doped helium nanodroplets. The process was accompanied by massive fragmentation. The neutral droplets contained, on average, 10^6^ helium atoms plus a hydrogen cluster with an unknown statistically distributed number of H_2_ near the center of the droplet. Ionization proceeds mostly by formation of He^+^ near the surface of the droplet, followed by resonant-charge hopping and, ultimately, charge transfer from He^+^ to the dopant.^26^, ^28^, ^32^, ^37^, ^38^ The energy released in the last step equals the difference in the ionization energies of He and the dopant. Subsequent ion–molecule reactions affect the energy balance further. Specifically, the energies for some likely reactions are [Eqs. (2 a)–(2 c)]:,^39^

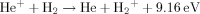
2 a

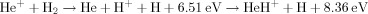
2 b

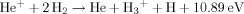
2 c

Note that the numerical values refer to gas-phase reactions. The energy of the product ions decreases upon solvation in helium, that is, the exothermicities increases.

At any rate, the reaction energy is of the order of 10 eV, 10^4^ times the cohesive energy of bulk helium (0.62 meV per atom). Thus, even though massive loss of helium is energetically possible, the energy would not suffice to evaporate all helium atoms from the doped droplet. However, charge localization and subsequent electrostriction in large helium droplets may result in the ejection of the dopant ion, solvated in a much smaller helium cluster.^37^, ^40^ The remaining excess energy in the ejected minicluster suffices to evaporate all or nearly all helium atoms.

Although the helium environment may reduce the extent of intramolecular and intra-cluster fragmentation of the dopant, it rarely eliminates it.^28^, ^32^ Mass spectra of, say, argon or krypton clusters^30^, ^41^ are virtually identical for clusters embedded in helium, or bare clusters. Neither the cluster sizes, or so-called magic numbers^42^ at which anomalies in the ion yield are observed, nor the extent of the anomalies, are affected. The evaporation of monomers which enhances the ion yield of magic clusters is not quenched by the presence of helium.

However, if any helium remains attached to the observed ions their temperature is necessarily low. Simply put, the vibrational temperature *T* of a cluster ion correlates with the dissociation (or evaporation) energy *D* of its most weakly bound constituent. For clusters with some 10 to 100 units one has [Eq. (3)]:

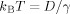
3
where *γ* is the Gspann factor whose value is, with few exceptions, near 25.^43^, ^44^ For H_*x*_^+^ with some ten or more helium atoms attached, *D* is a few meV, hence the temperature of the cluster ion is a few kelvins. The measured size distributions of these very cold He_*n*_H_*x*_^+^ ions reflects the size dependence of the evaporation energy *D*.

The He_*n*_H^+^ ion yield showed an abrupt drop from *n*=13 to 14, suggesting a similarly abrupt drop in the evaporation energy. This conclusion agrees with the drift-tube study by Kojima et al. who attributed the enhanced stability of He_13_H^+^ to a structure in which a strongly bound HeH^+^ is surrounded by 12 He atoms in an approximately icosahedral arrangement.^25^ However, the postulated structure is at variance with more recent theoretical work which shows that the ionic core is a covalently bound linear centrosymmetric He_2_H^+^.^14^–^17^ The next four helium atoms added to this core reside at equivalent sites in the plane perpendicular to the He_2_H^+^ axis. The calculated evaporation energy, corrected for the zero-point motion, remains nearly constant at ≍25 meV as *n* increases from 3 to 6, then drops by nearly a factor two for *n*=7.^16^ The drift-tube data suggested a magic number for *n*=6, although less pronounced than for *n*=13.^25^ The distributions in Figure 1 a reveal an abrupt change of the slope at *n*=6, consistent with the closure of a first solvation shell around an He_2_H^+^ core.

One atom added to He_6_H^+^ still showed a propensity to reside in the equatorial plane but as the cluster grew larger, the preferred number of atoms in the first shell was four.^16^ Thus, the magic He_13_H^+^ is best described as a two-shell system with seven atoms clustered around He_6_H^+^. However, all helium atoms outside the He_2_H^+^ core were floppy, the system exhibited increasingly non-classical behavior.^17^ Unfortunately, He_14_H^+^ has not yet been investigated theoretically. It remains to be seen if the evaporation energy does, indeed, strongly drop from *n*=13 to 14 as suggested by the experiments. It is worth mentioning that our data (Figure 1 a) suggest no further magic numbers, at least not below *n*=30.

Kojima et al. observed He_*n*_H_2_^+^ for 3≤*n*≤10 if H_3_^+^ was injected into the drift tube. However, injection of H_2_^+^ did not produce these ions.^25^ The drift-field dependence could not be evaluated because the signal was too weak. It is possible that the evaporation energies of the ions are too small for them to survive at the drift-tube temperature of 4.4 K. We observe He_*n*_H_2_^+^ up to *n*=30. Evaporative cooling in vacuum always cools, in principle, cluster ions to a temperature [see Eq. (3)] at which their evaporation rate equals the inverse experimental time scale.^44^ As discussed in the Section 2, the anomaly observed in the series of He_*n*_H_2_^+^ at *n*=13 has no counterpart in the series of deuterated cluster ions. It is impossible to tell which of the two distributions is affected by contamination. Statistically speaking though, a contaminant is more likely to feign an anomaly than to accidentally eliminate a genuine one. Thus, the apparent anomaly at He_13_H_2_^+^ should be treated with caution.

Trapping of helium and other noble gases at H_3_^+^ has potential astrophysical implications. H_3_^+^ is ubiquitous in the universe because it forms at the Langevin rate in reactions of H_2_^+^ with H_2_. It plays a pivotal role in interstellar chemistry.^21^ It is abundant in dense as well as diffuse molecular clouds and plays a crucial role in star formation. It has been suggested that H_3_^+^ may act as a sink for noble gases in protoplanetary disks and giant planets.^22^, ^45^ The computed evaporation energies for the first three helium atoms bound to H_3_^+^ are nearly constant at 45 meV. The He atoms in He_3_H_3_^+^ are bound at equal distances to the apexes of the triangular H_3_^+^.^24^

The steep rise in the ion yield that we observe in this size range (Figure 2 c) precludes any conclusion concerning He_3_H_3_^+^, instead we observed a pronounced magic number at *n*=12. Clusters larger than He_3_H_3_^+^ have not been investigated theoretically, but calculations for complexes of H_3_^+^ with argon suggest that larger complexes have a planar Ar_3_H_3_^+^ core.^23^, ^45^ Thus, it is not likely that the magic He_12_H_3_^+^ has icosahedral structure. Kojima et al. concluded from the drift-field dependence of the He_*n*_H_3_^+^ ion yield, which extended up to *n*=13, that *n*=9 and 10 are magic numbers.^25^ Their conclusion does not contradict our data (Figure 2 c) which exhibit strong drops from *n*=9 to 10, and 10 to 11. Kojima et al. did not list He_12_H_3_^+^ as magic, but mentioned that at a field of 1 V cm^−1^, the lowest value for which measurements were recorded, the size distribution “seemed to increase again at *n*=12 after passing a minimum at *n*=11.” We conjecture that *n*=12 was missed as a magic number in the field dependence because the measurements couldn′t be extended to fields lower than 1 V cm^−1^.

Finally we discuss the rather large ion yield of He_*n*_H_2_^+^ (Figure 1 a). Several other studies of helium droplets doped with diatomic molecules including N_2_,^26^, ^32^ O_2_,^32^ NO,^32^, ^46^ and CO^32^ have been reported. The studies agree that the diatomic monomer ions are reluctant to retain any helium, whereas their van der Waals clusters are readily observed with helium attached. Two mechanisms have been held responsible for the differences, namely the availability of low vibrational frequencies in the clusters that provide better coupling to the helium droplet, and the existence of another, more efficient cooling channel in clusters, namely evaporation of monomers.^32^ In contrast, our data show a very different trend. The yield of the diatomic monomer ion, He_*n*_H_2_^+^, is one to two orders of magnitude higher than that of ions containing H_2_ clusters (i.e. ions that contribute to the two groups at nominal mass 56 and 58 u in Figure 1 a). Perhaps the difference between our results (for H_2_) and those of other researchers (for N_2_, O_2_, NO, and CO) arises from differences in the size of the helium droplets which was about 10^4^ or less in previous studies,^26^, ^32^, ^46^ two orders of magnitude smaller than herein. Future experiments should investigate the effect of droplet size as well as the average number of dopant molecules in the cluster on the ion yield of He_*n*_H_2_^+^.

## 4. Conclusions

We have recorded mass spectra of hydrogen- and deuterium-doped helium nanodroplets ionized by electron impact. He_*n*_H_*x*_^+^ ions and their deuterated counterparts were mass-resolved up to 120 u. All conceivable combinations of *n* and *x* were observed. Magic numbers in the ion yield of He_*n*_H_1_^+^ are consistent with previous experimental and theoretical studies. The He_*n*_H_2_^+^ series, which has not been studied previously in any detail, exhibits a weak anomaly at *n*=19. The ion series is rather intense, in contrast to experiments with other diatomics molecules. He_*n*_H_3_^+^ shows a pronounced magic number at *n*=12 which had been missed in an earlier experimental study. Given that the chromophore in these ions is probably a planar He_3_H_3_^+^, the structure of the magic He_12_H_3_^+^ cannot be easily surmised.
